# Life Stressors in Young Americans are Linked via Asymmetric U-Shapes to Mental Health Severity

**DOI:** 10.21203/rs.3.rs-9464871/v1

**Published:** 2026-05-12

**Authors:** Dmitry Scherbakov, Nina de Lacy, Olga Barg, Jihad S. Obeid, Alexander V. Alekseyenko

**Affiliations:** Medical University of South Carolina; University of Utah; University of Pennsylvania School of Medicine; Medical University of South Carolina; Medical University of South Carolina

**Keywords:** NLSY97, depression, anxiety, life-course, machine learning, accumulated local effects

## Abstract

**Objective:**

To assess whether adolescent and young adult life stressors predict midlife depression/anxiety symptoms.

**Methods:**

Using The National Longitudinal Survey of Youth 1997 (n = 8,984; ages < 35 exposures; ages > 35 outcomes), we modeled maximum scores on CES-D/GAD-7 scales with stratified random forests and visualized via accumulated local effects. The U-shaped association was confirmed with quadratic linear regression.

**Results:**

Lower income, more cohabitations, earlier marriage, and ≥ 6 children predicted ≥ 1-point higher symptoms; many predictors (income, education, children, marriages) demonstrated variations of U-shaped association with the outcome.

**Conclusion:**

Early stressors were associated with midlife mental health symptoms, demonstrating non-linear patterns, which are often not identified by traditional statistical analysis.

## INTRODUCTION

Depression and anxiety remain prevalent mental health concerns [1, 2], with well-documented disparities across socioeconomic, racial, ethnic, and gender groups [3, 4]. Stressful life events are recognized as significant mental health factors [5]. Among those, adverse childhood experiences (ACEs) have long-lasting impact and affect outcomes later in life [6]. Less is known about gender and race-based differences in these experiences. A potential limitation of ACE studies is that they often focus on the childhood period (< 18 years), and do not cover early adulthood [7].

This study leverages longitudinal data from the National Longitudinal Survey of Youth 1997 (NLSY97) [8] to investigate how various life experiences pertaining to income, family structure, and relationship dynamics affect depression and anxiety symptom severity. The CES-D (Center for Epidemiologic Studies Depression) score [9] and GAD-7 (Generalized Anxiety Disorder 7-item) [10] serve as the primary measures of mental health symptoms in this analysis. We used random forest, a machine learning method capable of capturing non-linear relationships in data without specifying them beforehand [11].

By identifying key predictors and how they interact with the outcome across sex (female/male) and two income groups (high/low income), this associational study aims to inform strategies for prevention and intervention. The supplemental analysis presents results from analogue models stratified by race and prior mental health status instead of sex.

## METHODS

### Data Source

The NLSY97 survey study provides comprehensive multi-wave longitudinal data on a representative sample of U.S. individuals born between 1980 and 1984.

### Variable selection

Life stressors were selected by mapping NLSY97 variables to domains from the Stressful Life Events Schedule (SLES) [12]. The following domains were included: education, money (income), reproduction (number of children), romantic relationships, and miscellaneous events (deaths). Supplemental Table 1 Lists the variables selected for analysis.

### Variable Adjustments, Imputation, and Missing Values Handling

Variables from NLSY97 were processed as follows, median imputation was used for missing values in the predictors [13]:

- **Outcome: CES-D/GAD7 Score (0–21).** Maximum of CV_CESD_SCORE, GAD7_SCORE variables, with individuals with missing scores in all three variables excluded from analysis.
- **Personal income (continuous)**: Mean across selected survey years [13]. The mean value reflects the average dollar value across 2007–2022, which is lower than the 2025-dollar value due to inflation.
- **Personal income (dichotomized)**: Mean of income across selected survey years, dichotomized as Low (< $30,000) and High (≥ $30,000).
- **Education Level**: Replaced with an ordinal scale ranging from 0 (none) to 7 (“Professional degree (DDS, JD, MD)”).
- **Number of Children**: Sum of the two variables (CV_BIO_CHILD_HH_2015, CV_BIO_CHILD_NR_2015), reflecting all biological children respondent had up until 2015.
- **Age at Dating Debut**: Calculated from first reported sexual partner, cohabitation, or marriage year minus birth year.
- **Age at First Marriage**: Year of first marriage minus birth year.
- **Number of Relationship Breakdowns**: Sum across years.
- **Number of Cohabitations**: The last of the sequence of cohabitation partner numbers as reported in this categorical variable at any survey year/month selected for analysis.
- **Number of Marriages**: The last of the sequence of marital partner numbers as reported in this categorical variable at any survey year/month selected for analysis.
- **Number of Deceased Close Relatives**: Sum across selected years.
- **Sex at Birth**: No processing applied, but only used two sexes – female and male.

### Machine Learning Analysis

Two random forest models (for low and high income), implemented via the *caret* package in *R* with 5-fold cross-validation to select the *mtry* parameter, predicted CES-D scores with all other variables as predictors in the model. As the goal was associative rather than predictive, no hold-out test set was used; performance was assessed via cross-validation. Accumulated local effects grouped by sex were computed using the DALEX package and a bootstrap of 100 iterations to estimate the spread of the predicted values and confidence intervals. Results were visualized using ggplot2.

A similar separate analyses looked at 1) racial disparities using three random forest models (for Black, Hispanic, and Non-Black/Non-Hispanic cohorts, provided as Supplemental Fig. 2); 2) by prior mental health risk (High vs Low risk) defined as early depression (CES-D score in years 2000 and 2002) score > = 4.5 and neuroticism (2-items from Ten-Item Personality Inventory Scale, TIPI: calm/emotionally stable and anxious/easily upset) score > = 2 as “High risk”, and early depression < = 3 and neuroticism < = 2 defined as “Low risk” (Supplemental Fig. 3).

### U-Shape statistical test

To confirm that the relationship between predictors and outcome forms a U-shape, we fitted separate quadratic linear regression for each predictor and checked whether the quadratic term is statistically significant after Holm adjustment for multiple comparison.

## RESULTS

**Figure 1.**
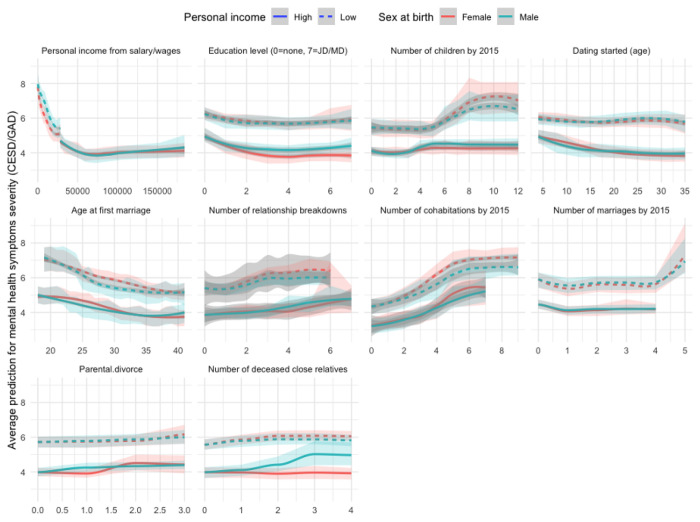
Accumulated local effect (ALE) profiles from the two random forest models for low income population (dashed line) and high income (solid line), grouped by sex at birth (red for female), with life stressors as predictors and CES-D/GAD maximum score in later life as outcome (displayed as predicted score points on y scale, with possible values from 0 to 21, where 21 is the highest symptom severity – only subset of values is shown to improve figure readability). Spline smoothing applied. Shaded areas represent bootstrap-based uncertainty in the accumulated local effect estimates across 100 resampled datasets.

7,314 out of 8,984 individuals had a non-missing CES-D/GAD score. The accumulated local effects profiles of the random forest models for females and males across 100 bootstrap iterations are presented in Fig. 1. At the optimal *mtry*, cross-validated performance (RMSE/MAE/R^2^) was 4.28/3.21/0.05 for the high-income model and 5.22/4.13/0.05 for the low-income model. We tested personal income, education level, number of marriages, and number of children for a U-shaped association with the outcome, and in all respective models found quadratic terms (responsible for curvature) statistically significant.

When comparing the results of the two models based personal income (dashed vs solid lines across all facets) persistent difference of approximately 2 points in CES-D/GAD score is seen: higher personal income was associated with reduced CES-D scores across all predictors. Furthermore, income as a predictor in both models showed a clear gradient: low earners had substantially higher symptom severity (over 3 points vs. the $60–$80k range), while very high earners (> $150k) had slightly elevated CES-D scores than the middle-income group. Education displayed another shallow U-shape—both very low and very high levels were linked to small increases (< 0.5 points), though among higher-earning women, more education reduced scores by about 1 point. Family size also followed a U-pattern: participants without children had modestly higher CES-D/GAD scores, scores were lowest with 1–3 children in high income group or with 1–5 children in low income group, while families with ≥ 6 children experienced higher symptoms by almost2 points in low-income households.

Dating before age 20 predicted roughly a 1-point increase in symptoms among high-income participants, whereas later first marriage was protective (≈ 2 points lower in low-income; ≈1 point in high-income). More relationship breakdowns added ≈ 1 point. Cohabitation count was a strong adverse marker, particularly for women (≈ 2.5-point increase). Marriages showed a U-shape: never-married individuals had ≈ 0.5-point higher scores; multiple marriages similarly resulted in elevated symptoms, with low-income participants showing ≈2-point increases at high counts. Parental divorce had minimal impact, and bereavement modestly raised scores, with a steeper ≈1-point rise for high-income men.

Supplemental Fig. 2 shows that the above patterns are consistent when prediction is stratified by race/ethnicity. Supplemental Fig. 3 shows similar patterns in analysis grouped by prior mental health risk, confirming that results hold even when accounting for earlier depression and neuroticism.

## DISCUSSION

Our study identified differences of 1–2 points in CES-D/GAD score based on analyzed life variable While this might appear minor at a population scale, it can nonetheless represent a clinically important difference [14].

We observed a shallow U-shaped income–mental health curve: symptoms were highest near poverty and rose slightly at very high incomes (< 0.5 points), patterns that linear models often miss [15]. Low income appeared more detrimental for men, with some elevation at very high incomes, consistent with social comparison effects [16]. Education was generally protective [17], but among low-income individuals, very high education predicted higher symptoms, plausibly reflecting effects of under-/unemployment [18]. Family size also resembled a U-shape, consistent with resource-strain mechanisms [19] [20].

Later dating debut and later marriage suggests a protective effect (without implying causality), aligning with evidence that early marriage can be an adversity [21, 22]. Yet very late marriage may increase divorce risk, and marital dissolution is linked to higher likelihood of depression [23, 24]. Relationship breakdowns predicted symptoms more strongly than bereavement counts; this pattern persisted after adjusting for early symptoms and neuroticism, though NLSY97 lacks reliable adolescent depression measures. Cohabitation and marriage counts showed similar associations with later symptoms; marriages followed a U-shape, with never-married status functioning as adversity. Prior work underscores the importance of early relationship histories [25], links higher partner counts and relationship transitions to elevated depression [21] [26], and identifies bereavement as a major risk—especially for females—while also refining these links (e.g., breakups predict depression chiefly when ties lack emotional commitment, are early versus peers, or are socially embedded; stable cohabitation may not increase depression) [27] [28] [29].

Parental divorce was not a strong predictor of adversity here despite meta-analytic findings about resource disruption [30]. Racial/ethnic stratification broadly mirrored these patterns across White, Black, and Hispanic groups, with nuances: early marriage (< 25) related more strongly to elevated midlife symptoms among White participants, and increases in cohabitations had stronger effects for White and Black participants [31].

## Limitations

We did not include all SLES domains (e.g., crime/legal, housing, employment, broader social ties, comorbid health issues) in our analysis. A limitation of the random forest approach is that it adapts closely to the specifics of the observed data, which may limit the generalizability of the results beyond the study sample. Other imputation methods besides the median could have been used [13].

## Conclusion

Socioeconomic conditions, family size, relationship timing and instability, and bereavement shape midlife depression/anxiety, often nonlinearly. U-shaped associations were common, underscoring the value of machine learning models, such as Random Forest, that capture curvature in the data.

## Supplementary Material

This is a list of supplementary files associated with this preprint. Click to download.

• APPENDIX.docx

## Data Availability

NLSY97 data are available via the U.S. Bureau of Labor Statistics National Longitudinal Surveys (https://www.nlsinfo.org). Analysis code and derived materials will be shared upon reasonable request.
